# The experiences of people with haemophilia and their families of gene therapy in a clinical trial setting: regaining control, the Exigency study

**DOI:** 10.1186/s13023-022-02256-2

**Published:** 2022-04-04

**Authors:** Simon Fletcher, Kathryn Jenner, Luke Pembroke, Michael Holland, Kate Khair

**Affiliations:** 1 Oxford Haemophilia and Thrombosis Centre, Oxford University Hospitals NHS Foundation Trust, Oxford, OX3 7LE UK; 2 Haemnet, London, N15 3JR UK

**Keywords:** Haemophilia A, Haemophilia B, Genetic therapy, Decision making, Informed consent, Clinical trial

## Abstract

**Background:**

Gene therapy has the potential to change the life experience of people with haemophilia and family members. Few studies have sought to explore the impact of gene therapy on both individuals and families. The aim of this study was to capture real-life experiences of gene therapy in People with haemophilia and their families.

**Results:**

Sixteen participants with severe haemophilia (11 haemophilia A, five haemophilia B), mean age 41.4 years (range 23–75 years), took part in a single qualitative interview; ten were accompanied by a family member. Mean time since transfection was 3.56 years (range 1–10 years). Participants saw their involvement in gene therapy as a positive experience, freeing them from the personal burden of haemophilia and furthering treatment options for the wider haemophilia community. However, participants reported being unprepared for the side effects of immunosuppression. Some also reported feeling unsupported and having little control over what was happening as their factor levels became the focus of the process.

**Conclusion:**

The results suggest that strategies need to be put into place to enable PwH fully to understand the process of gene therapy, and thereby make an informed choice as to whether it is a treatment they might wish for themselves. These include early and ongoing education, increased provision of psychosocial support and ongoing qualitative research.

**Supplementary Information:**

The online version contains supplementary material available at 10.1186/s13023-022-02256-2.

## Background

Haemophilia affects 1:3333 men worldwide [1], resulting in recurrent joint and muscle bleeding leading to joint arthropathy, muscle contracture and significant disability [2, 3]. The treatment of affected individuals involves the prophylactic replacement of the missing factor, which reduces the incidence of spontaneous bleeding events and resultant joint damage [4, 5]. Replacement therapy has improved life expectancy and quality of life of people with haemophilia (PwH), though limitations such as high costs and the treatment burden of frequent intravenous infusions remain [6, 7]. The latter has decreased with the development of extended half-life factor replacement products and factor VIII (FVIII) mimetics [8–10]. With the development of a number of gene therapy platforms for both haemophilia A and B, a potential cure also appears to be ever closer.

Gene therapies for haemophilia currently use an adeno-associated virus to insert the gene of interest (B domain deleted FVIII or factor IX [FIX] Padua) into hepatocytes, which then begin to produce the relevant clotting factor [11–14]. In the UK, 31 individuals (22 haemophilia A, nine haemophilia B) have so far undergone gene therapy in clinical trials examining the safety and efficacy of the technology [15]. Once biotechnology companies receive authorisation for their gene therapies [16] gene therapy may become a standard of care [17].

Qualitative studies have begun to explore the reasons why PwH might wish to consider gene therapy [18–20]. Some have sought to examine the impact gene therapy has had for those in clinical trials [21, 22], but none has considered the nature and impact of gene therapy itself and the immediate follow-up care it requires. While follow-up processes and requirements may change as gene therapy moves from clinical trials to a standard of care for haemophilia, many are likely to remain similar, including the need to monitor liver enzymes and factor levels and the need for immunosuppression. Without a clear understanding of the experiences of PwH who have had gene therapy, those who opt to have it in future and the haemophilia treatment centres that provide it will not truly understand the potential implications and may be ill prepared to deal with them.

**Fig. 1:**
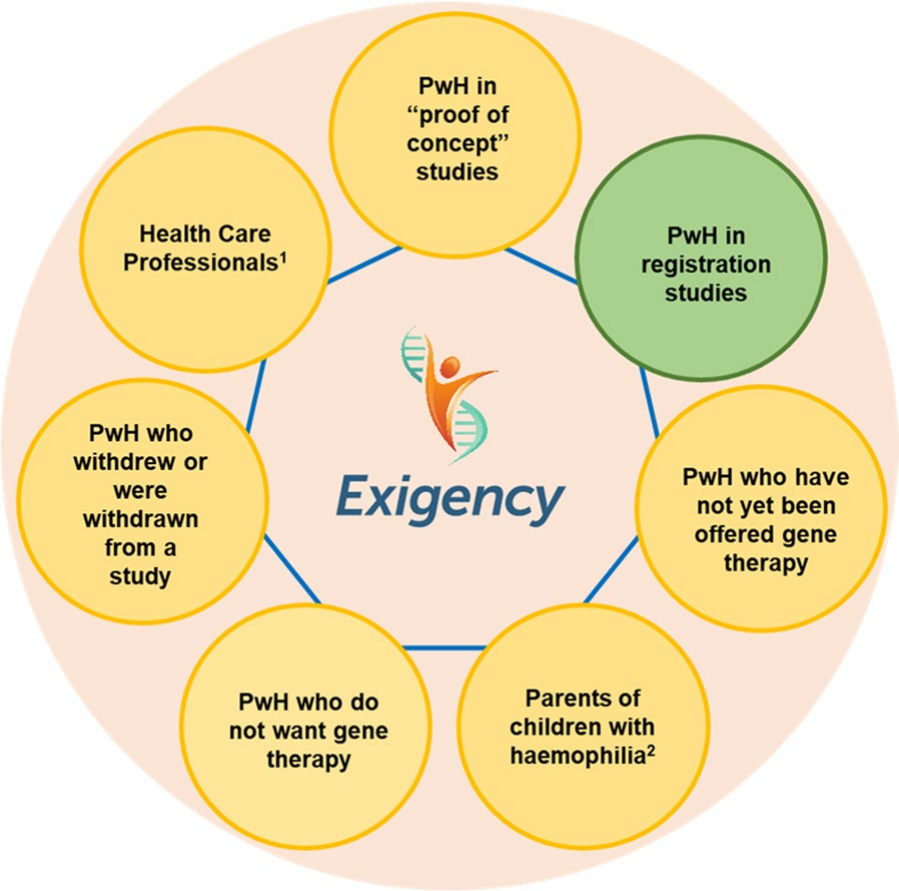
Exigency diagram

The Exigency programme was designed to explore the knowledge, expectations and experiences of gene therapy among a range of stakeholders in the UK haemophilia community (Fig. 1). This sub-study assesses the experiences of men with severe haemophilia who have undergone gene therapy in clinical trials. It is the first investigation by a team not involved with or affiliated to a gene therapy dosing centre.

## Results

### Sample characteristics

We invited 27 PwH (87.1% of those known to the UKHDCO) who had undergone gene therapy in the UK to participate. Sixteen PwH (51.6%) were interviewed along with 10 family members. Eleven participants had haemophilia A and five haemophilia B. The mean age of participants was 44.1 years (range 23–75 years). The mean time since gene therapy transfection was 3.56 years (range 1–10 years) and the mean self-reported factor level at the time of the interview was 0.33iu/ml (range < 0.01–1.37iu/ml). Three participants had been in phase 1 safety studies; the others had participated in subsequent phase 3 safety and efficacy studies. Recruitment was discontinued after 16 interviews as data saturation had been achieved. Three participants were known to SF and Six to KK prior to taking part in the study. None were known to both. For participant data see Table 1.

**Table 1:** Participant demographics

Trial number	Age range	Factor prior to gene therapy	Current factor level*^•^	Haemophilia type	Years since gene therapy treatment^†^	Immunosuppression required
Exi01	45–54	EHL	1.37	Haem A	1	Yes
Exi02	18–24	EHL	0.21	Haem A	1	Yes
Exi03	25–34	SHL	0.50	Haem A	1	Yes
Exi04	45–54	EHL	0.90	Haem B	1	Yes
Exi05	25–34	SHL	0.11	Haem A	5	Yes
Exi06	25–34	SHL	0.05	Haem A	3	Yes
Exi07	35–44	SHL	0.06	Haem A	1	Yes
Exi08	35–44	SHL	0.02	Haem B	9	No
Exi09	75–84	SHL	0	Haem B	10	No
Exi10	65–74	SHL	0.25	Haem A	2	Yes
Exi11	35–44	SHL	0	Haem B	10	No
Exi12	25–34	SHL	0.07	Haem A	3	Yes
Exi13	55–64	SHL	0.13	Haem A	2	Yes
Exi14	25–34	SHL	0.40	Haem B	4	Yes
Exi15	35–44	SHL	1.20	Haem A	1	Yes
Exi16	25–34	EHL	0.06	Haem A	3	Yes

*iu/ml

^•^Self-reported figures

^†^Whole years at time of interview

### Overview of findings

Four major themes emerged from the interviews: altruism, side effects of immunosuppression, control, and liberation.

### Altruism

All participants spoke of their reasons for wanting to take part in gene therapy. Nine spoke of their desire to help future generations of PwH.

“I’ve done it for the next generation. I don’t want anyone to have to go through what I went through.” [Exi06]

“One of the big factors of moving it forward was, of course, our daughter being a carrier, because clearly, from our point of view, it was all about if by the time she gets to the point of having a family and fate rolls the dice and she has a haemophiliac then wouldn’t it be amazing if someone went, ‘That’s not a problem’.” [Exi11]

This was especially true for those who had participated in the phase 1 studies, who knew they would only see minimal increases in their factor levels.

“I don’t want to sound like I’m a saint because I’m not a saint – but I felt I ought to give something back.” [Exi09]

For others the primary reason for trial participation was more personal; they were seeking a cure for themselves.

“I think to be a cure for me, to be honest.” [Exi12]

“I did [it] for my own little kind of mental state and my ability to be able to do things.” [Exi06]

### Side effects of immunosuppression

Thirteen participants required immunosuppression (eleven haemophilia A and two haemophilia B) either prophylactically, to prevent transaminitis, or to treat a transaminitis that occurred. The mean length of time on immunosuppressive therapy was 16 weeks (17.9 weeks haemophilia A (range 6–36 weeks) and 21 weeks haemophilia B (range 6–36 weeks)), with some requiring multiple courses of therapy. Ten participants and six family members stated immunosuppression and its side effects were the worst part of the gene therapy experience. One participant described the experience as “*absolutely horrendous*” [Exi06]*.* Another said he would only think about having gene therapy again (if the technology reaches a point where redosing is possible) if he was certain he would not have to have immunosuppressive therapy:

“[If they said], ‘You could have this gene therapy again, you don’t need to go on steroids, we’ve found another drug you can do that, will do the same, there’s no real side-effects,’ I would probably take it again.” [Exi03]

Both participants and family members described insomnia (n = 7), anger (n = 5) and feelings of depression (n = 2).

“I did not sleep. I didn’t need to.” [Exi06]

“I felt like it wouldn’t take much for me to flip out at someone, so I’d think, ‘If I just keep myself to myself, then I can’t upset anybody.’” [Exi02]

“That was a real dark, depressed… after a couple of weeks on them. I was angry, I was just… I broke down.” [Exi03]

Six participants said they had received immunosuppression for longer than they had expected and four had needed multiple courses.

“It was longer than I thought it was going to be for. I thought… I remember being told it would be six to eight weeks.” [Exi02]

“So, yes, in this next course of immune suppression – this is like chapter three of the immune suppression, the immune suppression diaries – that was the most intense time, for sure.” [Exi07]

While the overwhelming response to immunosuppression was negative, four participants reported some positive effects.

“Once I started taking the steroids and the tacro[limus] I felt quite good […] I had the sort of… the rush of blood to the head sort of energy of steroids.” [Exi07]

Others reported relief from their usual hay fever (n = 1) and relief from pain caused by arthropathy (n = 4):

“My inflammation that I keep getting in my joints or my muscles just did not happen at all for one month. So, I felt extremely healthy.” [Exi16]

For a full list of side effects experienced see Table 2.

**Table 2:** Side effects of immunosuppression

Side effects of immunosuppression*	Number of participants experiencing symptoms
Weight gain	7
Insomnia	7
Anger	5
Mood swings	3
Shaking hands	3
Hypersensitivity	2
Raised blood sugars	2
Depression	2
Pressure of speech	1
Constipation	1
Mania	1

*Self-reported symptoms

When asked to reflect on participation in gene therapy, all participants said it had been worthwhile, including those who now had no appreciable factor expression and were back on factor prophylaxis.

“I’d say yes, but just be prepared, really. Because it sounds really, really good – and it is good when it works – but you’ve got that period where – well, not for everyone – where it could be not very nice. Just be prepared for that, really.” [Exi02]

### Control

Half of the participants (n = 8), reported a need to control their haemophilia and its effect as important.

“It’s a bit difficult for somebody who’s not affected by the haemophilia to understand that you have to be able to control your life, and the home treatment was something that changed my life beyond all recognition. It allowed me to hold down a full-time job, which otherwise I would not have been able to do. It allowed me to go out of the house. It allowed me, or facilitated me rather, gradually overcoming my psychological fear of the world.” [Exi09]

For some, this search for control involved pushing boundaries of what was ‘allowable’ or ‘advisable’ to see what they were capable of. Four participants said this was important to their own sense of identity and wellbeing, although they admitted it had also led them to ignore their haemophilia and caused more harm than good.

“I think I’ve probably only just recently calmed down a little bit more. I was definitely the one that… I would… I put my body through probably more than I should have.” [Exi07]

“I’d had a really difficult probably three years of my life, with probably my physical and mental health, I suppose. And the haemophilia, I got really, really neglectful and I ended up… I ended up in hospital, very unwell.” [Exi05]

Half of the participants (n = 8) reported that rather than gaining control they had lost both control and individuality as they became subject to study-specific requirements.

“It was just everything for the results, and the blood tests and everything were more important than anything.” [Exi03]

“I suppose I’m saying that it’s the protocols that treat you as a number rather than the people.” [Exi04]

Some participants (n = 4) and family members (n = 2) felt this meant many of their concerns and issues were neither recognised nor adequately responded to.

“There was naturally stuff happening throughout the trial that I was noticing, and I was recognising and trying to have a conversation with them about – and it was like just falling on deaf ears.” [Exi05]

“Looking back, I’m starting to question a bit more why was I not just taken off that treatment the minute I expressed the level of discomfort that I was feeling.” [Exi07]

Two participants and their family members felt that mental health concerns were particularly poorly dealt with.

“Like, anything around mental health or psychological wellbeing was just like, nah… they did not want to know about that.” [ExiF03]

“I felt like at the time the trial was more important, the results of the trial were more important than [husband’s] mental health. I don’t think we really had the support for his mental health at the time.” [ExiF05]

Three participants thought some short-term loss of control was inevitable due to the constraining nature of study protocols. Four felt they had to wrest back some level of control, which took the form of refusing to attend appointments, weaning immunosuppression more quickly than advised, or refusing to have further courses of immunosuppression.

“They told me to prepare for it, because basically my liver enzymes kept rising and my factor’s been on a consistent downward slope. So, there was that time where… I think they said to me if I didn’t go on… Because they wanted me to go on immune suppression a fourth time and I said no. I said, ‘I can’t… for my own physical and mental health, and for my partner’s mental health, I don’t think we can go through that, so I’ll take my chances.’” [Exi07]

### Liberation

Despite the issues discussed above, the majority of those interviewed (participants, n = 12; family members, n = 3) described gene therapy as life changing.

“I can do most of the physical actions that I couldn’t do before. I can work in the garden, I can easily carry heavy bags from the grocery shop… And I don’t have to worry that my elbows or my shoulder joint or anything like that will just give me a bleed. So, it’s a peace of mind.” [Exi15]

“It’s unbelievably life-changing. Life-changing.” [ExiF08]

For others (n = 3) their improvement was down to ease of travel (not have to take large volumes of factor with them and navigate customs with needles and syringes) or the ability to participate in sports in ways not previously open to them.

“I play golf twice a weekend, I carried a bag five and a half miles, swung a golf club, and I never had a single problem. I’d get back and be completely fine. I wouldn’t even dream of doing that when I had haemophilia.” [Exi06]

Fourteen participants, including those in the early safety studies, had experienced a rise and then a decline in their factor levels. Four were on a prophylactic factor therapy regimen at the time of their interviews: two had returned to baseline levels of < 0.01iu/ml and two were experiencing bleeds despite having a factor level > 0.01iu/ml. The remaining 12 were not receiving factor replacement and 11 had not had any factor replacement therapy since transfection.

Of the 12 participants not currently on prophylaxis, all were aware there was a possibility of their levels dropping and that, at some point in the future, they may need to restart factor treatment, though there was hope this would not happen.

“I’m hoping that it comes down to such a level that I actually don’t need factor anymore at any time in the future.” [Exi01]

One participant thought gene therapy had “*not really made much difference*” [Exi03], as it was not able to fix the problems he had with his joints. He felt that if he had had it at age 18 “*it would have been probably a different story*”.

Further supporting quotes can be found in Additional file 1.

## Discussion

A growing number of studies have sought to examine the impact of gene therapy on the lives of individuals who have undergone the procedure [21, 22]. Most have focused on the positive results, many of which were also seen in this study, including ‘liberation’ from their condition and the worry of bleeds, the ability to participate in sports in ways previously not open to them, and to holiday without worrying about taking factor with them. The nature of the questions asked in a number of these studies have, however, been leading, guiding participants to talk about certain predefined negative aspects rather than those that were important to them.

Previous studies have also been undertaken by research teams involved in the dosing of the participants, which is a concern. There are well documented ethical concerns about unequal power relationships in clinician-led research, including coercion and bias, as participants can feel indebted to the interviewers and therefore inhibited talking about concerns they have [23–26]. A strength of our study is that neither of the interviewers worked at any of the dosing sites, and although several participants were known to one or other of the interviewers, none were known to both.

There are clearly many positives to gene therapy, but this study has highlighted a number of concerns that have not been described elsewhere, with the side effects of immunosuppressive therapy being the most difficult and troubling element. Although not seen in all cases, post vector infusion transaminitis is a recognised side effect of gene therapy [27, 28]. The underlying pathophysiology of this inflammation, and why some individuals are affected and others not, has not yet been fully described [29, 30]. However, even moderate rises in transaminase levels are associated with dramatic falls in factor expression [13, 31]. Many gene therapy studies have therefore included the use of immunosuppression, either prophylactically or reactively, in an attempt to prevent this [29]. The duration of immunosuppression required is not fully understood and, as has been shown in this study, can vary between individuals.

Immunosuppression is associated with significant safety concerns due to the side effects profile of the medications, including weight gain, hypertension, hyperglycaemia, altered mood, muscle spasm, neuropathy and psychiatric reactions [32]. Many of these were reported by participants in this study. The use of immunosuppression and perceived pressure from research staff to continue immunosuppressive treatment, despite side effects, meant some participants felt they were losing control rather than gaining it. There was recognition and understanding that this pressure existed due to concerns that factor levels could drop, but a feeling that maintenance of expression became the primary focus for research staff and that other questions and concerns were ignored or downplayed. Four participants felt self-advocacy was the only way to regain control and took themselves off immunosuppression sooner and more quickly than study teams advised. The need for control (over individuals’ lives, conditions and the research process) has been described in other studies [33].

Lack of psychosocial support, including lack of recognition of the need for it, was perceived by a number of participants as a concern. Provision of psychosocial support has been an ongoing concern within the UK haemophilia treatment community, with two thirds of comprehensive care centres and most haemophilia treatment centres having little or no access to services [34]. While access to support services is a wider issue, the concerns raised by the interviewees suggests that there should be greater emphasis on psychosocial needs, and that this should be integral to the package of care if gene therapy is to become a standard therapy. Psychosocial needs should also be acknowledged by the biotechnology companies running gene therapy studies and supportive measures incorporated into trials.

Future recipients of gene therapy, either in clinical trials or through licensed treatment must fully understand the therapeutic goals, the processes involved, and potential side effects. Known and unknown complications should be discussed alongside mitigation strategies that might be necessary. Consent to treatment should therefore be a process rather than an event, particularly as it is not possible to discontinue treatment once the vector has been given. This information process should begin in childhood and continue throughout life [35, 36]. In this way, when PwH decide that gene therapy is something they wish to receive, they will have a greater understanding and expectation of the process and potential outcomes.

## Limitations

This study involved a self-selecting, UK-based sample of participants with ready access to prophylactic haemophilia treatment prior to their gene therapy. There could therefore be an inherent, unintended selection bias in this group. This bias has however been mitigated to a degree by the size of the sample (> 50% of the UK gene therapy cohort).

Data saturation usually requires 20–25 individual interviews [37, 38] but there is a degree of consistency in this study due to the homogeneity inherent in the gene therapy participant group. As no new codes or themes emerged in interviews 15 and 16, the research team felt that data saturation had been achieved. There may be a greater diversity of opinion and experience as gene therapy becomes more widely available, and it will be necessary to continue to interview future recipients and family members to continue to understand what affect it has.

The Exigency programme [19, 35] has been carried out in a high-income country where PwH have good access to intensive treatment. The concerns and issues raised may differ from those of low- and middle-income countries, or the emphasis placed on them may be different. Further research needs to be undertaken to delineate a greater understanding of these concerns. We believe it is important that such studies are undertaken by groups not linked to any single dosing centre to avoid researcher bias, thereby enabling participants to voice their concerns without fear that their comments could upset the teams looking after them.

## Conclusion

When it becomes more widely available, gene therapy for haemophilia may become a standard of care, potentially changing the face of future haemophilia care. If this is to happen and is to be seen as a safe and attractive treatment, PwH need a greater understanding of the processes and implications of the therapy, some of which have been highlighted in this study. Strategies including early and ongoing education, and the adequate provision of psychosocial support throughout the process should be established. Ongoing longitudinal qualitative research will be needed to understand what impact gene therapy for haemophilia has throughout all life stages.

## Methods

### Study design

A qualitative interview study was conducted with men with severe haemophilia who had undergone gene therapy in the UK. Interviews were undertaken between 1 August 2020 and 31 August 2021.

The interviews followed an interview guide based on a review of the literature and the experience of the study team (see Additional file 2). Questions addressed the individual’s haemophilia and treatment history, the decision-making process of opting for gene therapy, and their experience of gene therapy.

### Recruitment and data collection

Participants were recruited through haemophilia centre referral, social media, and word of mouth referral. All participants took part in a single 1 h interview conducted by two researchers (SF and KK) via the video conferencing platform, Zoom®. Participants were given the option to be interviewed with a family member. The initial recruitment target was 25 individual interviews though recruitment could be discontinued at the discretion of the researchers if data saturation was achieved, or further recruitment was unlikely. The latter condition was added as UK data show that just 31 PwH have received gene therapy [16].

### Analysis

Each interviewee was randomly assigned a study number (PwH, Exi01-Exi16; family members, ExiF01-ExiF10). All interviews, which were recorded and transcribed verbatim, were facilitated by SF and KK who each have more than 30 years’ experience in nursing. Transcripts were thematically analysed by both researchers after each interview using inductive coding (SF: NVivo® for Mac; KK: manual coding). Prior to each scheduled interview the researchers met to discuss, review and refine emergent codes, enabling their exploration in subsequent interviews. On completion and analysis of the final interview, the researchers met to discuss all transcripts, further refine codes and identify final themes.

## Supplementary Information

**Additional file 1.** Supporting Quotes.

**Additional file 2.** Interview Guide.

## Data Availability

The datasets generated and/or analysed during the current study are not publicly available as it contains un-anonymised participant information. Data sets are available from the corresponding author on reasonable request.
