# Supplementary material for: The experiences of people with haemophilia and their families of gene therapy in a clinical trial setting: regaining control, the Exigency study
Source: Orphanet J Rare Dis. 2022 Apr 4;17:155. doi: 10.1186/s13023-022-02256-2 (PMC8981747; doi:10.1186/s13023-022-02256-2)
Supplement: Supplementary file 1 — Additional file 1. Supporting Quotes. [file 13023_2022_2256_MOESM1_ESM.docx]

| Additional File 1. Quotes Document | |
| --- | --- |
| **Altruism** | |
| Exi01 | It’s seeing the sort of thing that my cousin went through, and hearing the stories of what he went through from my aunt and from my mum. I thought there is no way that I want any child to have to go through that or to go through what I had to go through when I was younger. So, if I can help in any way, I will glad do so. Because the more information we have, the better it is and, essentially, the more people it will be able to reach. |
| Exi05 | And then I read a bit about it and I thought… Well, I suppose it was the girls as well. [Brother] said to me, “Mate, the girls are going to be carriers. It’s only a generational thing, so you might as well try and help out.” And I’m all about helping out, I’m all about the people. I do it in my job. I’m all about social care and standing my ground. So he changed my mindset. |
|  | And I thought, “Oh, I can help the girls, I can help that.” So, I did it for everyone else. But I haven’t, really. If you ask me now, I’ve done it for me. And I’m glad I have done it for me. |
| Exi06 | Yes, and it’s things like that. Like, at the time, [brother] had daughters and they’re going to pass it on and things like that, so… It’s doing the next… doing your bit to help out the next person. Always. |
|  | As I said at the beginning, I didn’t do this for me, I’ve done this for the next people. |
| Exi09 | I wanted to give something back, as well as help future generations. |
| Exi10 | If you don’t try it, no one else benefits. Somebody’s got to be bold enough. |
| Exi11 | Clearly, there were risks, as the big dossier that we had to read and sign up to which then said, “If you do this, you could die.” But beyond that, I just thought, well, the risk was worth it to put medicine one step forward in terms of delivering effectively a lifelong treatment for haemophilia – which was always my goal. |
| Exi12 | I’m proud of myself because I tried to help others. |
| Exi13 | I’m certainly glad to contribute to research. I’m glad, I’m always glad to step up and do that or any other… any other benefit people can take from it, any lessons that they can learn, absolutely. Yes, absolutely. And I’d be happy to volunteer again on the basis that people might be able to learn something from it. And, obviously, a massive personal gain at the same time. |
| Exi16 | If something were to happen, you are still contributing to science, you’re contributing some extremely valuable data, and I’m glad to be part of this. |
| ExiF01 | It’s actually a lot… it’s actually… I’ll be honest with you, to me it’s a wonderful thing that he’s done and that he continues to do. |
| ExiF05 | It’s looking at the future for us, isn’t it. What can [husband] do to get a better future for our children’s children? That was our main thing. |
| ExiF07 | Yes. I think, for me, the fact that it’s taken this time and it is going to be available, given our daughter and her prospect of having children at some point, it’s like… actually, if she does have children, there is that chance that there is treatment that’s going to be available which will make her life and the child’s life a lot easier. But yes, I suppose I was proud of you having done it, absolutely, because you put your life on the line, really, to do it. It was a big risk. Although I tried not to think about it at the time in that way, because I knew how much you wanted to do it. |
| **Immunosuppression** | |
| Exi01 | And from then it’s been, yes, we’ve had the rough points of having… but I think that’s mostly prednisolone, that’s actually not been the trial, the gene therapy. That’s actually been… the side-effects have been the prednisolone. |
| Exi02 | But I felt maybe like I excluded myself a little bit from people, because I felt angry all the time. I felt like it wouldn’t take much for me to flip out at someone, so I’d think, “If I just keep myself to myself, then I can’t upset anybody and no one’s going to upset me,” if that makes sense. |
|  | Yes, I gained a lot of weight. Yes, that’s probably the main ones. And then the tacrolimus, the biggest thing that I didn’t like about the tacrolimus was how much my hands shaked. |
|  | That’s hard because I really didn’t enjoy the pills…Yes I probably would [*have gene therapy if it was possible*], yes. |
| Exi03 | Yes, the steroids. Because I had to go on steroids. That was… They put me on them after about six or seven weeks, and they said, “There’s signs of something, so you need the steroids to get the liver going.” And they took me off them for about three weeks and shoved me back on them again. |
|  | I think in the end I went out on my bike. But I was still getting angry with cars just going through… because they were going to slow and I was just flat out for an hour and a half, just to try and burn some energy off. |
|  | That is one thing I’ve said to them: “You’ve got to find a different way from doing steroids.” |
|  | If they said that “you could have this gene therapy again, you don’t need to have steroids, we’ve found another drug you can do that will do the same, there’s no real side effects,” I would probably take it again. |
| Exi04 | You could stay on the steroids for a positive reason, and you can, because you’re going to the gym and it was great; the arthritis in your feet you didn’t really feel, you just didn’t notice it. |
| Exi05 | I was so grumpy, so grumpy. I had to get up, do all these samples, then get in the car. And I had to eat – I don’t like eating in the morning and then you had to eat before you have this tablet. And then the steroids were a whole other… |
|  | It’s just… I don’t know. Just no understanding of it. And I suppose when I was going there and showing them my sleep chart, going, “This is how much I’m sleeping,” and they were going, “Yes,” it was like… It got to a point, it was like, “I don’t know if this is funny anymore because I’m feeling manic. I’m feeling genuinely, genuinely manic.” Like, I’m going out for runs at three o’clock in the morning; the neighbours think I’m nuts. |
| Exi06 | I probably slept 40-50 minutes a night for maybe five or six weeks and would run like I’d slept 12 to 14 hours. Non-stop, brain goes, body’s on the go, just… yes. I couldn’t stop myself. |
|  | Oh yes, terrible mood swings. |
|  | I took the steroids for a good… at least three months, probably four. Yes, it was a long time, it was a long time. And I think when I got off the steroids, I think that was the moment when my body tried to catch up with itself and I… yes, I was just like, “Whoa, I need to sit, I need to sleep, I need rest. My body can’t continue.” |
| Exi07 | But then almost overnight, taking steroids, my ankle pain was pretty much… I wouldn’t say it was gone, but it was certainly eased. |
|  | I had this hypersensitivity in my hands. |
|  | I crumbled. I was actually in tears, I was crying, because I was in so much pain. And it was literally just like that [*mimes gentle touching*], but it was like an electric shock through my body. |
| Exi10 | I think it was probably six weeks. I don’t recall being on it that. |
| Exi12 | I used to wake up night-time, because I had McDonald’s next to the house where I live, I used to jump in the car, go to McDonald’s, buy two or three burgers, eat them at the one time. I used to wake up in the morning, have a big, large, extra-extra-large… which I never have because I’m 55 kilos. So, imagine, since that time I’m 60, 61, 62, sometimes even 63. I used to eat a lot. I used to eat like maybe ten times a day. |
| Exi13 | I was kept awake at night, I was very buzzy during the day, but I never felt tired because I was so buzzy, so it wasn’t a terrible reaction. |
| Exi14 | Yes, so I was lucky there weren’t any side-effects. |
| Exi15 | It’s all those immunosuppressants, all those steroids… They really affect you. You have to be prepared for that. Really, you have to be very strong-minded. You have to be set up that you want to do it, because it’s very easy to break down, especially when your mood changes. I felt… one time I felt, “Oh, come on, I have to… I think I’ll have to break it, I’ll have to stop it, because I don’t want to do it anymore,” those immunosuppression things – shaking hands, trembling hands were killing me, they definitely were killing me. |
|  | I think seven months. Yes, I think seven months, as far as I remember. |
|  | Because definitely I wasn’t feeling like I was inside my own body. My body reacted completely differently, completely. Like, the trembling and shaking, that’s nothing compared to the hypersensitivity – in your fingers, in your feet, on the skin… It was a nightmare. To be honest, it is a nightmare. Taking a shower, for example, hypersensitive skin, taking a shower on immunosuppression, you feel like you’re burning. And it doesn’t matter if the water is cold or it’s warm or it’s hot. Just water dripping on the skin and you feel like you’re burning. It was a nightmare. But as I said… I don’t know, maybe because I was so excited to do it and I was just… That was my goal. Maybe my mind was set up only on this and that’s why I just went through with it. |
| Exi16 | It’s hard to explain, but when I was on steroids, I probably had the best time of my life. |
| ExiF02 | [Partner]: So, six or seven months. ExiF02: It felt like longer. |
|  | He got angry about it all the time, saying, “I regret it. Why did I do this stupid thing?” all the time. |
|  | I don’t think he looked at the bigger picture; he looked at it right there and then. Because I kept saying to him, “Just think, in so many months’ time you’ll be haemophilia-cured,” and he was like, “No! It’s not worth it!” |
| ExiF03 | And you, a few times, burst into tears because you didn’t know what you… how you were going to react. |
|  | So, it was a really dark time. |
|  | As soon as you came off the steroids you were back to normal. |
| ExiF04 | Never slept. And then was mad into exercise, which is so nice because then he had all the happy endorphins on top of it. So, it was like a mega-ride for a few months, the excitement… And also, he ate, which is… you know, quite… [husband] is really a sporadic eater, but you just wanted to eat all the time. |
|  | You didn’t sleep. You were literally like wired. I mean, his eyes were like whooo the whole time. I liked it because it’s the most active you’ve ever been in your whole life. Like, you were literally just buzzing around the house. |
| ExiF05 | Horrible. |
|  | I hated it. |
| ExiF06 | You were eating so much, you know, just the amount of food that we kept in the house, it just vanished |
|  | And I think also social situations as well, you started avoiding social situations because you were quite worried about going out and being uncomfortable around people, so things you would say. I think that you slowly started avoiding… |
| **Control** | |
| Exi03 | Yes. Anything against the product wasn’t… it was chucked out the window. |
|  | When I go up there, it’s not the haemophilia team, as such, that’s seeing me; it’s the research team. And I feel like they’re more working for the drug company than the haemophilia side of people, which could be half the sort of problem, because they were… |
|  | Yes, I got through it and I said, “Look…” I managed to… I said, “Look, I can wean off” – I needed to wean myself off them. And I did it a bit quicker than they wanted to and they were saying, “No, don’t do that,” they wanted to do it a different way, and I was like, “No, I’m doing it this way.” And I did it my way instead of their way. I said, “I’m not going back on them.” I said, “If we go back on them, that’s it – no more.” |
| Exi04 | Part of me is going to say you’ve certainly treated me as an individual. But then you have also treated me as a number, for the simple reason being that when one of the other guys went through what he did, it was assumed that I was going to go through what I did, so you treated me the same way. That, in my opinion, was treating me as a number. |
|  | The protocols would drive you to treat patients as a number. So, perhaps it’s that that you would need to look at. |
|  | I had it in my head, thinking to myself, “I’m not going on those bloody steroids again.” And then, at the same time, the other part of me is just saying, “Well, if I have to go back on the steroids I’ve got to go back on the steroids – there’s no two ways about it. |
| Exi05 | There was naturally stuff happening throughout the trial that I was noticing and I was recognising and trying to have a conversation with them about – and it was like just falling on deaf ears. Like, anything around mental health or psychological wellbeing was just like, nah… they did not want to know about that. |
|  | It’s so much more with your psychology and your mental health, and those emotions – they failed to recognise that. |
|  | There were bits of the trial that I thought, “You haven’t got an answer for that.” So, what I did in the end, I armed myself, I went and did the reading on everything that came out, and then I took that to them. |
|  | You know, unlike if I go in with a certain type of bleed, there’s A, B, C, D, E, F, G that we go through, whereas this is kind of somewhat unknown. So, I think I definitely afforded them the benefit of the doubt. |
|  | But now, with a bit of time, yes… Even the thing with my hands, I feel like now, looking back, I’m starting to question a bit more why was I not just taken off that treatment the minute I expressed the level of discomfort that I was feeling. |
|  | And at that point I said, “I can’t take this stuff anymore, I’m done. I’m not chasing that number anymore – it’s not worth it.” And they tried to convince… They didn’t want me to stop taking the tacrolimus at the hospital. I think I probably communicated it as “I’m done with this, I want off. I’m not… I’m not chasing that number anymore. I’ll go back to injecting.” I think I actually said to them, “I’ll inject every day for the rest of my life to not feel the way I feel now,” because it was just so intense. It was so… Yes, just that pain. |
| ExiF03 | I said to them, “He needs to come… he needs to reduce the steroids or he needs to come off them,” and they were like… He couldn’t, obviously, come off them and… But I was really concerned about his mental health and what he was going to do. |
|  | They weren’t interested in… I don’t think you could fix your mental health at the time, but they weren’t… They were more interested in the trial… |
| ExiF06 | So, I think… I don’t… It’s hard for me to say whether they acted rightly or not. I mean, I think there was a feeling throughout it when we’d kind of go… we felt like there’s this trial thing, they wanted to succeed, they want to learn more, they wanted to put him back on it so we can see what happens. But I think a lot of the time we felt like, “Hold on a minute – let’s just stop this thing and just take care of what [Exi13] is going through.” |
|  | But it’s important for me to say that perhaps you weren’t able to communicate what you were going through in the same way that you are describing it right now because of everything you’ve been through. |
| **Liberation** | |
| Exi01 | I discussed it with [Wife] and we both talked through it, and we said, “Right, we’ll go for it.” It’s five years, but it’s five years of a possible chance of – for want of a better word – being normal, or as normal as possible for a haemophiliac. |
|  | I keep on using the word ‘normal’, but it’s a case of I feel like a normal human again, for want of a better description, even though having haemophilia doesn’t make you any less normal than anybody else. It’s a different type of normal – chalk and cheese, really. |
| Exi02 | I’m fine with it. I’m happy with where I’m at. I don’t inject, I don’t get little niggles in my fingers that are bleeds, and I don’t… Just life’s easier. |
| Exi06 | So, I’d say gene therapy changed my life in a way that I never thought it ever would. So, I was always brought up on the pretence of I have an incurable disease; I inject myself with prophylaxis and that’s as close as I’m ever going to get to normal – and even at that point you’re not normal. But I have to say, gene therapy has probably proven me wrong. |
| Exi10 | But I was just thinking hopefully this is going to get rid of a lot of the problems for me. Sort of liberating, I think, was the feeling. And I think ultimately that is what it’s done – it’s liberated me. |
| Exi12 | Yes, to be honest. I’m not worried anymore. So now, for example, if I have a pain or… I know where I’m going to have the pain because the joints are damaged, but I know because of the haemophilia I’m 100% safe in that way. |
| Exi13 | It has been life-changing, and to have no treatment and still just be normal is just phenomenal. It’s hard to get your head around sometimes, just to forget about it. |
| Exi15 | Within a few days or one week, you realise that something changed, that you don’t have to worry about going out, you don’t have to worry about having factor VIII in the fridge, you don’t have to worry about waking early because you have to do the injections in the very early morning to be prepared to do some actions during the day. |
| ExiF03 | Because it has been life-changing in the main. |
