# Supplementary material for: The experiences of people with haemophilia and their families of gene therapy in a clinical trial setting: regaining control, the Exigency study
Source: Orphanet J Rare Dis. 2022 Apr 4;17:155. doi: 10.1186/s13023-022-02256-2 (PMC8981747; doi:10.1186/s13023-022-02256-2)
Supplement: Supplementary file 2 — Additional file 2. Interview Guide. [file 13023_2022_2256_MOESM2_ESM.docx]

| Additional File 2. Interview guide | |
| --- | --- |
| **Part 1. People with haemophilia who underwent gene therapy** | |
| Thank you for agreeing to take part in this project about how people feel about having undergone or considered undergoing gene therapy. Everything you tell us in this interview will be treated with complete confidence – your identity will never be revealed.  We know that people who have undergone gene therapy show better quality of life on questionnaires, but we want to know more about how gene therapy impacts on your day-to-day life.  Are you OK to proceed and are you happy that we record the interview? | |
| **Questions** | **Prompts** |
| Can you begin by telling me how old you are and a little bit about what hobbies you have? |  |
| Can you tell me about your haemophilia – when were you diagnosed? What treatment you were on before consenting to take part in the gene therapy study? |  |
| Can you recall how many bleeds you had in an average year before consenting to take part in the gene therapy study? |  |
| How are your joints? Do you have any joints that bleed more than others? How did you manage those before and has that changed now? |  |
| Have you ever had an operation in hospital? What was it for? |  |
| Now I’d like to talk about your reasons for choosing to take part in the gene therapy study and what influenced your decision. | - When and how did you first hear about gene therapy? - Who instigated the idea of gene therapy – was it you or your clinicians? - Can you tell me about the tests you had to go through to take part in the study? - Did you feel that you were given enough time and information to make your mind up about taking part? - How long did the process take? - Did you feel fully informed throughout this time? - What were you thinking during this time? - What did you feel when you finally found out you were eligible to undergo the gene therapy? |
| Can you tell me about the actual therapy and what you went through as part of the therapy? | - Did you find any of the procedures embarrassing? - Did you find the frequency of visits difficult? - Did you find any of the post therapy requirements difficult? |
| Now that you have had the treatment can you describe to me the impact that it has had on your life   - Imagine a scale of 1 to 6, where 1 is no impact and 6 is the highest impact. How much of an impact on your life does your treatment represent?   o Now vs before | - How active are you on a day-to-day basis? Do you do any sports? - How have you adjusted to not having to treat yourself in the way you used to? - What has surprised you most since you had the gene therapy - What is it like to no longer have to treat yourself with prophylactic factor? Who taught you to do prophylaxis? What was it like? |
| What impact has having gene therapy had on you and your family? | - Has anything surprised you about the impact gene therapy has had on your life? - Have there been any negative impacts? - How worried are you about an injury? - Have you had any bleeds since having undergone gene therapy? - If yes, where were they? When did they happen? Do you know what caused them? What action did you take? How would you treat now vs. past? How did you feel about this bleed? - If no, then how does the prospect of a bleed make you feel? |
| What about pain? Do you have any pain now?   - Imagine a scale of 1 to 6, where 1 is very little pain and 6 is the worst pain. How bad is that pain? | - How has it been over the past month (do you get pain every day, is it joint related, is it haemophilia related?) - What did you usually do in the past when you experience pain related to your haemophilia? – is it different now? - How does arthritic pain differ from the pain of a bleed (if applicable)? - Is the pain different now? |
| What impact has gene therapy had on your time? |  |
| What are your hopes/expectations for gene therapy? What are your goals for the next 6 months? Do you have any concerns about it? |  |
| Have you heard about any other treatments that might be available in the future? |  |
| If the gene therapy stopped working, how would you feel about having to go back to your previous treatment? |  |
| What advice would you give to others considering having gene therapy? |  |
| Is there anything else you would like to say or ask of me? |  |
| **Part 2. Family participants of individuals who have undergone gene therapy** | |
| Thank you for agreeing to take part in this project about how people feel about having undergone or considered undergoing gene therapy. Everything you tell us in this interview will be treated with complete confidence – your identity will never be revealed.  We know that people who have undergone gene therapy show better quality of life on questionnaires, but we want to know more about how gene therapy impacts on their day-to-day life.  Are you happy to proceed and are you happy that we record the interview? | |
| Can you begin by telling me how old you are and a little bit about what activities you and your partner/spouse/child/sibling enjoy doing together? |  |
| How long have you known your partner/spouse? |  |
| How long have you known that your partner/spouse/child/sibling has haemophilia? |  |
| Did you have any idea what haemophilia was before this? |  |
| How different was the reality of your partner’s/spouse’s/child’s/sibling’s haemophilia from what you thought it might be? |  |
| I’d like to go on now to ask a little bit about your partner/spouse/child/sibling and how their haemophilia has affected both him and your family: | - What treatment was your partner/spouse/child/sibling on before he took part in the gene therapy study? - Can you recall how many bleeds your partner/spouse/child/sibling would normally have in an average year? - When your partner/spouse/child/sibling had a bleed, how did he manage them? |
| What difference has the gene therapy made to you partner/spouse/child/sibling? | - How long has it been since your partner/spouse/child/sibling underwent the gene therapy? - Has your partner/spouse/child/sibling had any bleeds since he has had the gene therapy? - Does he react to the bleeds he has now in a way different now than he did before having the gene therapy treatment? |
| How do you think the therapy has impacted on you as a family/carer? |  |
| Can you give me examples of what impact this has made? |  |
| Do you have any worries for the future? |  |
| If the gene therapy stopped working, how would you feel about your partner/spouse/child/sibling having to go back to his previous treatment? |  |
| What advice would you give to others considering having gene therapy? |  |
| Is there anything else you would like to say or ask of me? |  |
